# Clinical management of aortic valve regurgitation after left ventricular assist device implantation: prediction, assessment, and treatment strategies

**DOI:** 10.3389/fcvm.2026.1726345

**Published:** 2026-02-17

**Authors:** Sen Li, Yingjian Xu, Shiqi Zhang, Zhifu Han, Xiangyu Liu

**Affiliations:** Department of Clinical Research, Aerospace Taixin Technology Co., Ltd., TianJin, China

**Keywords:** aortic regurgitation, heart failure, left ventricular assist device, predictive factors, transcatheter aortic valve replacement

## Abstract

Left ventricular assist devices (LVADs) have become a core treatment modality for end-stage heart failure. However, aortic regurgitation (AR) remains a common postoperative complication that significantly threatens patient outcomes. This narrative review examines the pathophysiological mechanisms, predictive factors, assessment methods, and intervention strategies for AR following LVAD implantation, aiming to provide guidance for clinical practice. Studies have shown that preoperative age ≥60 years, female gender, lower body surface area, mild AR, proximal ascending aorta diameter/body surface area > 15.5 mm/m^2^, and higher cumulative dose of beta-blockers increase the risk of AR. Postoperative aortic valve opening restriction and prolonged LVAD support time are strong predictors of significant AR. Among device types, axial flow pumps have a higher incidence of AR than fully magnetic levitation centrifugal pumps; Traditional assessment methods have limited applicability and should be combined with hemodynamic characteristics (such as central venous pressure, pulmonary capillary wedge pressure, and pulmonary artery pulsatility index) and multimodal imaging techniques such as transthoracic echocardiography (AR width/LVOT width ratio), transesophageal echocardiography, and cardiac magnetic resonance imaging; Preoperative repair or replacement of moderate or severe AR can reduce postoperative risks, and transcatheter aortic valve replacement (TAVR) is the preferred treatment for significant AR postoperatively. Future efforts should focus on optimizing the assessment system and improving device design to enhance long-term patient outcomes.

## Introduction

**Figure 1:**
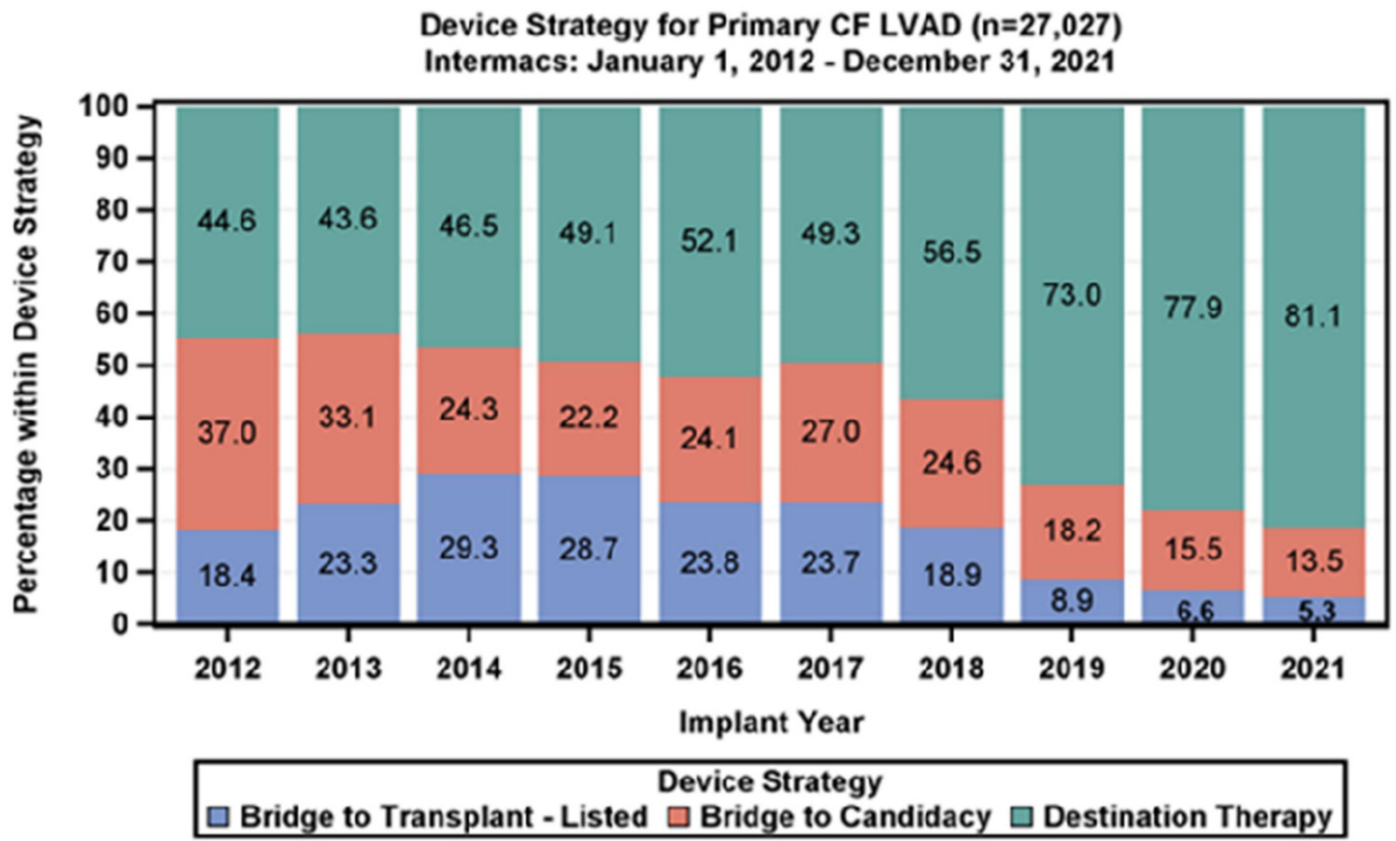
The proportion of DT treatment has increased year by year.

Heart failure is a highly prevalent chronic cardiovascular disease worldwide, and its incidence in China has been steadily rising with the acceleration of population aging (1, 2). Currently, non-pharmacological treatment options for heart failure include heart transplantation and left ventricular assist device (LVAD) support (3). Among these, heart transplantation was once considered the “gold standard” treatment for end-stage heart failure, significantly improving patient outcomes and quality of life. However, the scarcity of donor organs and surgical resource limitations have resulted in extremely low coverage rates, far from meeting clinical needs (4). Additionally, LVAD, as the core technology for mechanical circulatory support, has seen a steady increase in the application of destination therapy (DT), reaching 81.1% in 2021, as shown in Figure 1, making it an important alternative treatment option for end-stage heart failure patients (5).

However, as LVAD support time increases, the clinical risks associated with aortic regurgitation (AR) become increasingly evident. Following LVAD implantation, reduced left ventricular pressure leads to an increase in transvalvular pressure gradient across the aortic valve. Combined with the high shear stress generated by retrograde blood flow, this can trigger aortic valve remodeling and functional deterioration (6). This pathological change creates a “vicious cycle”—blood pumped into the aorta via the LVAD then flows back into the left ventricle through the incompetent aortic valve, significantly reducing effective cardiac output and leading to recurrent heart failure symptoms in patients. In severe cases, emergency heart transplantation may even be required (7). Studies show that the incidence of significant AR within one year post-LVAD implantation reaches 25%–30%, and is closely associated with increased patient mortality (8–10). Therefore, AR has a significant impact on patient prognosis, and its mechanisms and intervention strategies require further investigation.

The pathogenesis of aortic regurgitation (AR) is complex and involves multiple factors. Following LVAD implantation, pathological processes such as increased transvalvular pressure gradient, retrograde blood flow formation, local high shear stress, and aortic root tissue remodeling interact synergistically to promote the development of AR (7, 11). Additionally, the reduced pulse pressure gradient and weakened aortic pulsatility caused by the LVAD further accelerate the deterioration of aortic valve function (12). Studies have shown that aortic valve dysfunction is a serious long-term complication faced by LVAD patients, but there remains controversy regarding its pathophysiological mechanisms and treatment strategies.

Although the clinical significance of AR is well-established, there remains debate regarding its predictive models, assessment criteria, and optimal timing for intervention. This comprehensive review focuses on the predictive factors, assessment methods, and treatment strategies for AR following LVAD implantation, aiming to provide evidence-based guidance for optimizing clinical management to improve patient long-term survival rates and quality of life.

## Predictors of AR following LVAD implantation

The occurrence of AR following LVAD implantation is not a random event; its risk can be predicted through a combination of preoperative baseline characteristics and postoperative key factors. Identifying these predictors in detail can help clinicians develop early intervention strategies to reduce the risk of AR occurrence (Table 1).

**Table 1:** Predictors and risk factors for aortic regurgitation after LVAD implantation.

Category	Risk factors
Preoperative factors	Age ≥60 years; Female gender; BSA <2.0 m^2^; Mild AR at baseline; Aortic root diameter/BSA >15.5 mm/m^2^
Postoperative/Device factors	Aortic valve opening restriction; Prolonged LVAD support duration; Axial flow pumps (vs. Centrifugal)
Surgical factors	Anastomosis inclination angle ≥90°

### Preoperative baseline characteristics

Multiple studies have confirmed that preoperative clinical indicators can effectively predict the risk of postoperative AR. When analyzing clinical data from a large number of LVAD implant patients, some studies have examined the association between various preoperative clinical characteristics and the occurrence of postoperative AR. For example, multiple studies have shown that patients aged ≥60 years have a significantly higher risk of developing AR after LVAD implantation compared to younger patients, suggesting that age is an important influencing factor for postoperative AR (8, 13). From a physiological mechanism perspective, as age increases, the tissue structure of the aortic valve undergoes progressive degenerative changes, leading to reduced elasticity and compliance of the valve leaflets. Consequently, following LVAD implantation, the aortic valve is more prone to functional impairment in response to hemodynamic changes, thereby triggering AR (14).

Gender factors are also associated with the risk of postoperative AR. A retrospective study by Lauren K et al. based on the INTERMACS database showed that female gender is an independent risk factor for moderate-to-severe postoperative AR (13). Some studies speculate that this may be related to the cardiovascular anatomical structure and physiological characteristics of women, such as the relatively smaller size of the aortic root in women, which may make them more sensitive to hemodynamic changes under LVAD support, thereby increasing the risk of AR. This provides a potential anatomical basis for why women are more prone to AR after LVAD implantation (15). Additionally, a cross-sectional study of 438 patients with hypertension and left ventricular hypertrophy (including 266 women and 172 men) found significant correlations between aortic root dilation in women and cardiac output, mild aortic regurgitation (16).

Body surface area is also a key predictive indicator, with studies showing that a lower body surface area is a known risk factor for AR occurrence under LVAD support (8, 13). This may be because patients with smaller body surface areas have relatively smaller hearts and aortas, and after LVAD implantation, the hemodynamic balance within the heart is more easily disrupted, affecting the coordination of aortic valve opening and closing, ultimately leading to AR occurrence.

The preoperative status of the aortic valve plays a crucial predictive role in the occurrence of postoperative AR. A clinical study involving 316 patients who underwent LVAD implantation showed that at 1 year postoperatively, the proportion of patients without significant new-onset AR was 94.5% among those without preoperative AR, while the proportion was 64.6% among those with mild preoperative AR (17). Related studies have also indicated that even mild AR prior to surgery significantly increases the likelihood of AR worsening postoperatively (13).

Anatomical parameters of the aortic root play a significant role in predicting the risk of postoperative aortic regurgitation (AR). Studies have shown that when the proximal ascending aorta diameter/body surface area exceeds 15.5 mm/m^2^, the risk of postoperative AR significantly increases (18). Following LVAD implantation, the pulse pressure difference decreases, leading to changes in the mechanical stress on the aortic wall. This results in reduced elastin content and smooth muscle cell apoptosis in the aortic wall, causing an increase in the diameter of the aortic root within six months postoperatively. This root dilation disrupts the normal valve leaflet closure mechanism of the aortic valve, preventing complete closure of the leaflets and thereby triggering AR (19).

Additionally, preoperative medication use is also considered to be associated with the risk of postoperative AR. Imamura et al. (20) found that a higher cumulative dose of beta-blockers prior to surgery is also a predictive factor for significant AR postoperatively. Furthermore, studies have found that patients who have used beta-blockers long-term (defined as chronic use exceeding 60 days) have a higher absolute incidence of adverse cardiac events before adjustment compared to non-users, but their risk is reduced after adjustment, suggesting that long-term use is related to the condition of specific surgical patients and may reflect the association between cumulative dose and etiology and disease progression (21).

### Postoperative key factors

Postoperative factors are equally important in predicting the risk of aortic regurgitation (AR). The open state of the aortic valve is considered one of the strongest predictors of postoperative AR. In a study on exercise-induced aortic valve opening, researchers divided LVAD postoperative patients into an exercise group and a control group, with the exercise group receiving regular aerobic exercise training in the early postoperative period. The results showed that the frequency of aortic valve opening in the exercise group was significantly higher than that in the control group (22). Additionally, in patients receiving long-term LVAD support, aortic valve function gradually deteriorates over time, significantly increasing the risk of developing significant AR (23). Therefore, promoting aortic valve opening has become one of the clinical treatment strategies.

LVAD device-related factors significantly influence the occurrence of postoperative AR. Studies have shown that support duration is positively correlated with the risk of AR, meaning that as LVAD support duration increases, the incidence of AR also increases (8). Device type is also an important factor influencing the incidence of AR. Compared with fully magnetic levitation centrifugal pumps (such as HeartMate 3), axial flow pumps (such as HeartMate II) have a higher incidence of AR, and patient readmission rates also increase (24, 25). This may be related to the constant high-speed operation mode of axial flow pumps, which causes the valve to endure high mechanical loads over the long term, accelerating valve wear and functional deterioration.

**Figure 2:**
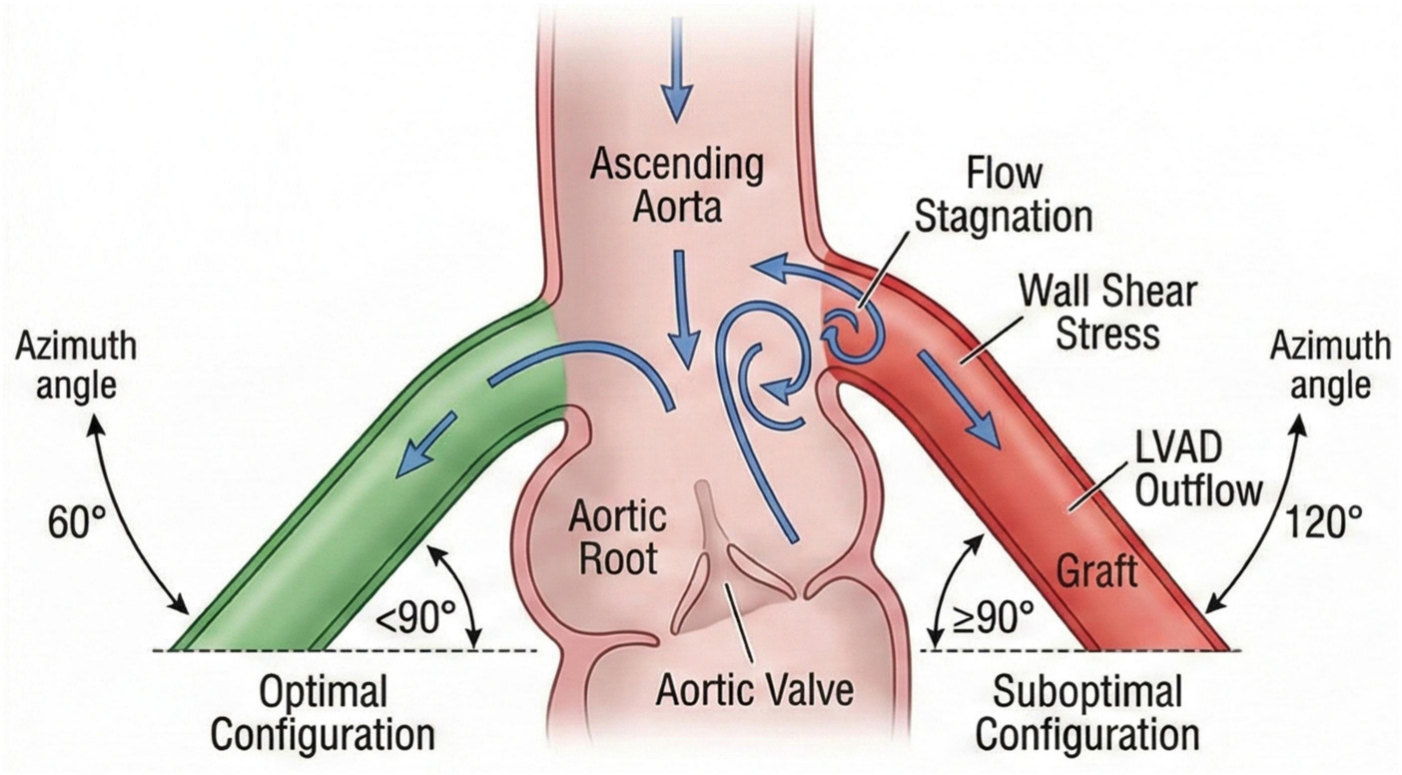
Schematic diagram illustrating the optimal anastomosis inclination angle (<90°) and azimuth angle for the LVAD outflow graft to minimize hemodynamic stress on the aortic root.

Among surgical technical factors, the angle of artificial vessel anastomosis significantly influences the risk of postoperative AR. *In vitro* simulation studies have shown that a lower anastomosis position, an inclination angle of less than 90°, and an azimuth angle of 60° or 120° help reduce blood flow stagnation at the aortic root and create normal wall shear stress and moderate pressure conditions in that region (26). Therefore, selecting a smaller anastomosis angle and a 60° azimuth angle to establish an optimal blood flow pattern is an important strategy for effectively reducing the risk of postoperative AR in LVAD surgery (Figure 2).

## Methods for assessing AR after LVAD surgery

After LVAD implantation, patients experience significant changes in their hemodynamic patterns, limiting the applicability of traditional AR assessment systems. Therefore, it is necessary to combine hemodynamic characteristics with imaging techniques to develop targeted assessment strategies for accurately determining the severity of AR and its impact on the circulatory system.

### Changes in hemodynamic characteristics

Following LVAD implantation, patients experience a fundamental shift in their hemodynamic patterns, presenting numerous new challenges and characteristics for the development and assessment of AR. Traditionally, aortic regurgitation primarily occurs during cardiac diastole, when blood flows back from the aorta into the left ventricle. However, under LVAD support, due to its continuous blood flow pattern, left ventricular pressure remains relatively stable and consistently below aortic pressure throughout the cardiac cycle. This unique hemodynamic state prevents the aortic valve from opening normally during systole, resulting in continuous regurgitation throughout the cardiac cycle (6). In an *in vitro* study based on CT imaging, Kassi et al. (27) developed a simulation model incorporating a HeartMate II axial flow pump. Their findings demonstrated that as pump speed increased, both net pump flow and retrograde regurgitant volume increased significantly. Unlike traditional diastolic regurgitation, LVAD patients may experience full-cycle regurgitation—increased pump speed causes left ventricular pressure to remain consistently below aortic pressure, resulting in the aortic valve remaining closed during systole (6).

Hemodynamic monitoring parameters are crucial in assessing the condition of AR patients post-LVAD surgery. Hemodynamic monitoring revealed that AR patients had higher central venous pressure (CVP) (11 ± 5 vs. 8 ± 5 mmHg, *P* = 0.03), higher pulmonary capillary wedge pressure (PCWP) (16 ± 6 vs. 12 ± 6 mmHg, *P* = 0.02), and lower pulmonary artery pulsatility index (PAPI) (2.3 ± 1.3 vs. 3.6 ± 2.4, *P* = 0.01) (28). These changes in indicators reflect the impact of AR on the circulatory system from multiple dimensions: increased CVP suggests increased right ventricular load, which may be related to left ventricular volume overload affecting right ventricular function through interventricular septal interaction (29, 30); Elevated PCWP directly reflects increased left atrial pressure, which is a direct consequence of AR-induced increased left ventricular preload (31); and decreased PAPI reflects reduced right ventricular drive capacity in the pulmonary circulation, associated with mismatched right ventricular afterload increase and improved left ventricular function post-LVAD implantation, serving as an early marker of deteriorating cardiopulmonary function (32).

### Imaging assessment techniques

Imaging techniques are key tools for assessing AR after LVAD implantation, with different methods offering distinct advantages, often used in combination in clinical practice (Table 2).

**Table 2:** Recommended echocardiographic parameters for assessment of AR in LVAD patients.

Modality	Parameters	Significance
TTE Parameters	AR jet width/LVOT width ratio; Pressure half-life (PHT); Vena contracta (VC)	TTE provides semi-quantitative assessment
TEE Parameters	LVAD inflow/outflow conduit position; Valve structure visualization	TEE is superior for structural analysis and excluding conduit artifacts

Transthoracic echocardiography (TTE) is the preferred imaging modality for assessing AR due to its non-invasive nature, convenience, and widespread application. Color Doppler imaging can be used to observe the characteristics of the regurgitant jet, and the severity of AR can be semi-quantitatively assessed using the ratio of regurgitant jet width to left ventricular outflow tract (LVOT) width (AR width/LVOT width) (33, 34). Additionally, measuring the left ventricular end-diastolic diameter (LVEDD) and end-systolic diameter (LVESD) can assess left ventricular volume load and functional status (35). For patients with continuous-flow LVAD (cf-LVAD), guidelines for assessing AR severity recommend a multi-parameter approach based on traditional TTE parameters, including pressure half-life (PHT), vena cava diameter (VC), the ratio of jet width to LVOT width, and confirmation of hemodynamic outcomes (27).

Transesophageal echocardiography (TEE) Compared to TTE, TEE is not affected by chest wall interference, providing clearer images and is suitable for patients with poor image quality or requiring high-precision assessment (36). Following LVAD implantation, TEE can be used to ensure adequate cardiac exhaustion and successful LVAD activation, while assessing the position and blood flow distribution of the LVAD inflow and outflow conduits. This information is critical for determining AR status, as abnormal conduit positioning may affect hemodynamics and thereby influence AR status (37). Additionally, 3D-TEE multi-plane reconstruction has been successfully applied in transcatheter mitral and tricuspid valve interventions, aiding in the formulation of treatment plans for related cardiac conditions (38).

Cardiac magnetic resonance imaging (CMR) is a well-established diagnostic technique for evaluating valvular heart disease and has become increasingly widespread in clinical practice in recent years. Its advantages include the provision of high-quality images without the use of ionizing radiation and, in some cases, without the need for contrast agents. CMR enables a comprehensive assessment of the heart, including biventricular function, left ventricular remodeling, myocardial fibrosis, and associated valvular lesions (39). CMR can accurately quantify AR and provide precise assessment of regurgitation through the acquisition of relevant data, offering reliable evidence for subsequent analysis and clinical decision-making (40). Additionally, CMR can assess left ventricular remodeling and function, detect myocardial fibrosis, and aid in prognosis determination; it can also display aortic root lesions, assisting in the development of personalized treatment plans (39, 41).

Computed tomography angiography (CTA) can automatically identify key anatomical landmarks of the aortic root and generate precise quantitative data such as aortic root diameter, aiding physicians in more accurately assessing aortic root conditions and providing data support for evaluating AR risk (42, 43). In summary, in clinical practice, multiple imaging techniques are often combined to achieve precise assessment of AR post-LVAD implantation, thereby optimizing treatment decisions.

## Treatment strategies for AR associated with LVAD implantation

### Preoperative intervention

AR significantly impacts the efficacy of LVAD treatment; therefore, assessing the severity of AR and implementing appropriate interventions prior to LVAD implantation is critical. For patients with mild or lower-grade AR, aortic valve intervention is typically not required; however, for patients with moderate or higher-grade AR, correction during LVAD implantation is recommended to optimize treatment outcomes and reduce postoperative complications.

Although concomitant aortic valve surgery may increase surgical risk, the benefits of aortic valve intervention typically outweigh the risks associated with LVAD failure (e.g., reduced pump performance) caused by significant AR postoperatively (44).

**Figure 3:**
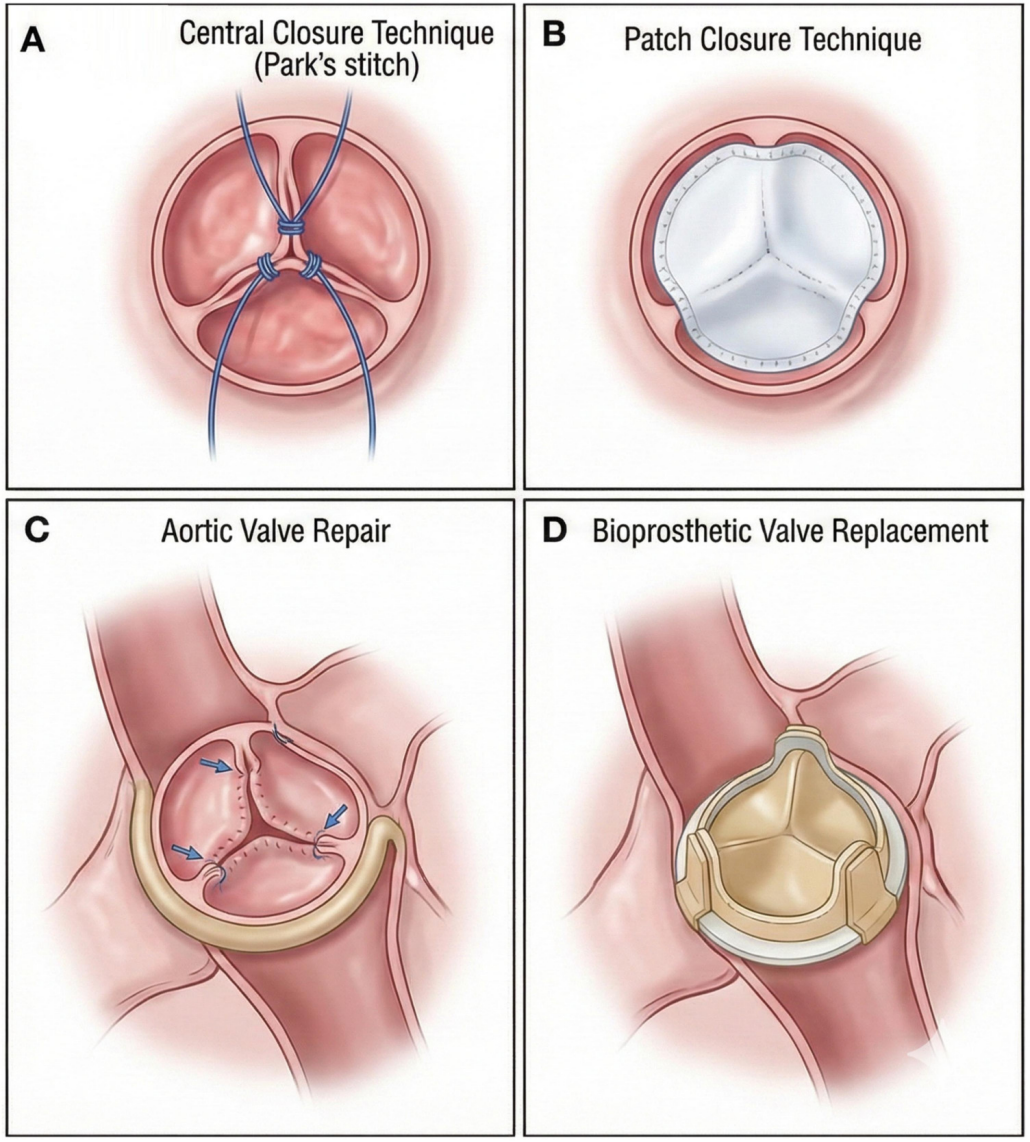
Illustrations of surgical techniques for aortic regurgitation (AR). **(A)** Central coaptation is restored using a central closure technique like the Park's stitch. **(B)** A patch is used to close a defect or perforation and reinforce the closure. **(C)** Repair of a prolapsing aortic valve cusp. **(D)** Replacement of the native valve with a stented bioprosthesis.

Based on the patient's specific condition, aortic valve treatment strategies can be divided into the following three types:

(1) Aortic valve closure: In certain specific cases, complete closure of the aortic valve may be considered to prevent its opening and thereby avoid the development of progressive AR postoperatively. Common surgical procedures include the use of pericardial patches or surgical suturing (45). For example, a new method involves using a polytetrafluoroethylene (PTFE) patch to close a mechanical aortic valve by filling the gap between the annulus and the leaflets and securing the valve in the closed position. The specific procedure involves cutting a low-porosity PTFE felt patch into four circles to create a double-layered gap-filling patch, which is then sutured and fixed to the mechanical valve to achieve valve closure (46). This method avoids circulatory circuit issues caused by aortic regurgitation (AR), such as cardiogenic shock, inadequate perfusion, and multi-organ failure, thereby improving the patient's hemodynamic status and quality of life (47). However, if the mechanical aortic valve is closed during LVAD implantation, there is a risk of sudden death if the LVAD ceases to function (48). Studies have shown that in acute myocardial infarction patients undergoing LVAD implantation, those with aortic valve closure have significantly lower survival rates compared to those with aortic valve repair or replacement (49). Therefore, aortic valve closure is not recommended as the primary surgical procedure.
(2) Aortic valve replacement: For patients requiring long-term LVAD therapy, aortic valve replacement is a common strategy. Bioprosthetic valve replacement is recommended, and mechanical aortic valves are not advised. Currently, bioprosthetic valve replacement is widely used in LVAD patients (50, 51). From a mechanistic perspective, patients under LVAD support have low-frequency or even prolonged non-opening of the aortic valve. If a mechanical aortic valve is selected, the lack of sufficient blood flow to wash the valve leaflets can significantly increase the risk of thrombosis (52, 53). If patients have previously received a mechanical aortic valve before surgery, it should be replaced with a bioprosthetic valve during LVAD implantation (48). Compared to mechanical valves, bioprosthetic valves have the advantage of not requiring long-term anticoagulation, which can further reduce the bleeding risk associated with anticoagulant therapy, especially for LVAD patients with additional high-risk factors for bleeding (54). It should be noted that bioprosthetic valves are prone to long-term degeneration, and over time, leaflet deterioration or fusion may occur, which might require additional interventions (55). Additionally, transcatheter aortic valve replacement (TAVR), as a minimally invasive treatment modality, is increasingly being applied in LVAD-related AR patients (56, 57).
(3) Aortic valve repair: When the aortic valve structure is largely intact, with only partial lesions such as leaflet prolapse or perforation, aortic valve repair is a suitable option. By repairing the diseased leaflets to restore the normal structure and function of the aortic valve, regurgitation can be effectively reduced (58). In AR patients, aortic valve repair and replacement have comparable perioperative outcomes, but the long-term reintervention rate is higher for repair (59). In a study of AR patients prior to LVAD implantation, patients who underwent aortic valve repair combined with LVAD implantation showed a significant reduction in AR severity during postoperative follow-up (60). Aortic valve repair methods include leaflet repair techniques (such as leaflet folding and leaflet perforation repair) and annulus reconstruction techniques (61, 62). Repair using an aortic valve annulus ring can reduce the incidence of AR by counteracting further expansion of the aortic root, and the procedure is relatively simple with favorable outcomes (63) (Figure 3).

### Postoperative management

Following LVAD implantation, effective management of AR is critical for patient outcomes. Treatment strategies are categorized into non-surgical and surgical approaches.

#### Non-surgical treatment

Optimizing anti-heart failure drug therapy aims to improve cardiac function, reduce cardiac workload, and alleviate the adverse hemodynamic effects caused by AR (64, 65). Angiotensin-converting enzyme inhibitors (ACEIs) or angiotensin II receptor blockers (ARBs) inhibit the renin-angiotensin-aldosterone system (RAAS), reducing peripheral vascular resistance, alleviating afterload on the heart, and decreasing left ventricular pressure, thereby helping to relieve AR-induced volume and pressure overload on the left ventricle (66). RAS inhibitors and statins As part of the secondary prevention treatment regimen for aortic valve regurgitation patients following aortic valve replacement surgery, RAS inhibitor therapy is associated with better long-term outcomes (64). Over 50% of heart failure patients require diuretic therapy after LVAD implantation. Furosemide has been used for over 50 years to relieve congestion in patients with fluid overload and is the most commonly used loop diuretic for hospitalized patients with decompensated heart failure (67, 68). However, it is important to note that excessive diuresis may lead to hypovolemia, impairing the normal pumping function of the LVAD. Higher diuretic doses prior to surgery are associated with an increased risk of early right heart failure following LVAD implantation (69).

Secondly, adjusting the pump speed and flow settings of the LVAD can optimize the patient's hemodynamic characteristics (70). Appropriate adjustment of pump speed can increase effective cardiac output and ensure adequate organ perfusion (70, 71). Flow regulation is equally important; adjusting the LVAD's flow settings can alter left ventricular filling and emptying states, affecting aortic valve opening and closing. Studies indicate that increasing LVAD flow can moderately elevate aortic pressure, aiding in reducing regurgitation, but may also lead to increased regurgitant flow (72). Beyond acute pump speed adjustment, preventative strategies focusing on intermittent pump speed modulation have gained increasing attention. The Lavare cycle, available in HeartMate 3 devices, periodically reduces pump speed to create transient pulsatility, facilitating aortic valve opening and promoting blood flow wash-out at the aortic root (73). This intermittent speed reduction helps prevent valve leaflet fusion and reduces thrombus formation on the aortic valve cusps, potentially mitigating the progression of AR in long-term LVAD support (74). Similarly, artificial pulse algorithms that periodically alter pump speed have been shown to increase the frequency of aortic valve opening events, which may preserve valve function and delay the onset of significant AR (75).

#### Surgical treatment

For patients who develop significant AR postoperatively or who fail to respond to medical therapy, surgical intervention should be initiated as early as possible. Surgical approaches are broadly categorized into two types: invasive surgery (aortic valve closure, repair, and surgical aortic valve replacement) and transcatheter aortic valve replacement (TAVR) (76). While invasive surgical techniques are well-established and effective, they carry a higher risk of re-thoracotomy for LVAD patients.

TAVR offers advantages such as minimal trauma and fewer postoperative complications compared to invasive surgery, making it more suitable for surgical treatment of significant AR in LVAD patients (57). It is important to note that TAVR was initially primarily used for patients with aortic valve stenosis, who typically have concomitant aortic valve thickening and calcification. Therefore, compared to patients with aortic valve stenosis, patients with AR may lack sufficient calcification to stabilize the valve, thereby increasing the risk of valve displacement (77, 78). The absence of annular calcification in LVAD patients with pure AR presents unique challenges for TAVR, as traditional valve anchoring mechanisms rely heavily on calcific deposits to secure the prosthesis (79). Self-expanding valves may offer advantages over balloon-expandable valves in this population due to their continuous radial force and ability to conform to non-calcified annular geometry, though careful sizing remains critical to prevent migration (80). Additionally, the enlarged aortic root in LVAD patients can lead to a larger annulus size, which may affect valve anchoring and increase the risk of paravalvular leakage (81, 82). Recent case series have demonstrated successful TAVR in LVAD patients using both self-expanding and balloon-expandable platforms, with careful pre-procedural CT planning essential for optimal valve sizing and positioning (83).

## Discussion

As a core alternative treatment for end-stage heart failure, the continuous increase in the proportion of “purposeful treatment” with LVAD confirms its clinical value (5). However, AR, as one of the most prominent complications after LVAD surgery, not only affects treatment outcomes but also directly threatens patient survival. Its clinical management complexity and specificity require further in-depth discussion (8). This paper discusses the clinical management challenges, mechanism associations, and optimization strategies for AR following LVAD implantation based on existing research evidence, providing guidance for clinical practice.

The pathophysiological mechanisms of AR following LVAD implantation exhibit a complex interplay of multiple factors. The reduced left ventricular pressure under LVAD support leads to increased transvalvular pressure gradients, combined with shear forces generated by retrograde blood flow, triggering valve remodeling, reduced elasticity, and functional deterioration. This “reflux-volume overload-deterioration of cardiac function” vicious cycle may reduce effective cardiac output, exacerbate heart failure symptoms, and even necessitate emergency heart transplantation (7, 84).

Accurate identification of high-risk populations is a prerequisite for optimizing AR clinical management. Existing studies have identified multidimensional predictive factors, encompassing preoperative baseline characteristics and postoperative key factors. At the preoperative level, independent risk factors include age ≥60 years, female gender, body surface area <2.0 m^2^, mild AR at baseline, proximal ascending aorta diameter/body surface area >15.5 mm/m^2^, and high cumulative dose of beta-blockers. Postoperative core factors include aortic valve opening restriction, prolonged LVAD support time, and device type (axial flow pumps carry higher risk). Additionally, surgical technical factors such as artificial vessel anastomosis with an inclination angle <90° and azimuth angle of 60° can reduce risk. However, current predictive models have limitations: existing studies are primarily based on single-center data and lack a unified risk scoring system; some factors (such as the mechanism of action of beta-blockers) remain poorly understood and may be confounded by patients' underlying conditions. Future studies should utilize multicenter cohort research to integrate clinical, imaging, and device parameters, develop dynamic predictive models, and achieve individualized risk stratification.

Changes in hemodynamic patterns post-LVAD implantation (such as full-cycle reflux) limit the applicability of traditional AR assessment methods, necessitating the establishment of a targeted assessment system. Hemodynamic monitoring shows that AR patients have significantly elevated central venous pressure (CVP) and pulmonary capillary wedge pressure (PCWP), while the pulmonary artery pulsatility index (PAPI) is reduced. These indicators can reflect the extent of circulatory system involvement at an early stage and assist in assessing the impact of AR on cardiac function. The combined application of imaging techniques is central to precise assessment. Transthoracic echocardiography (TTE) is the preferred method, using the AR width/LVOT width ratio for semi-quantitative assessment of regurgitation severity, though its accuracy is influenced by image quality; transesophageal echocardiography (TEE) clearly displays valve structure and catheter position, making it suitable for high-precision assessment; Cardiac magnetic resonance imaging (CMR) quantifies regurgitation, assesses myocardial fibrosis, and evaluates aortic root lesions, providing comprehensive information for prognostic assessment. In clinical practice, hemodynamic indicators and multimodal imaging should be combined to achieve a comprehensive assessment of “anatomy-function-prognosis”.

AR interventions should follow the principles of “individualization and staged treatment”, combining preoperative prevention with postoperative stepwise therapy. For patients with moderate or severe AR, valve repair or replacement is performed preoperatively: repair is suitable for lesions with intact structure; bioprosthetic valves are recommended for replacement, while mechanical valves are not advised. Aortic valve closure is only used as a life-saving measure due to the risk of sudden death. Mild AR can be improved by optimizing medications (e.g., ACEI/ARB to reduce afterload, diuretics to control volume) and adjusting LVAD pump speed. Significant AR is primarily treated with transcatheter aortic valve replacement (TAVR), but CTA assessment of the annulus size is required to reduce the risk of displacement and paravalvular leakage. The timing of intervention remains controversial and should be decided based on symptoms, hemodynamics, and imaging progression. Currently, the timing of intervention remains controversial: some studies advocate “early intervention” to prevent worsening heart function, while others believe that “symptom-driven” intervention is more cost-effective. Future randomized controlled trials are needed to clarify the intervention threshold and develop decision-making criteria based on patient symptoms, hemodynamics, and imaging progression.

**Figure 4:**
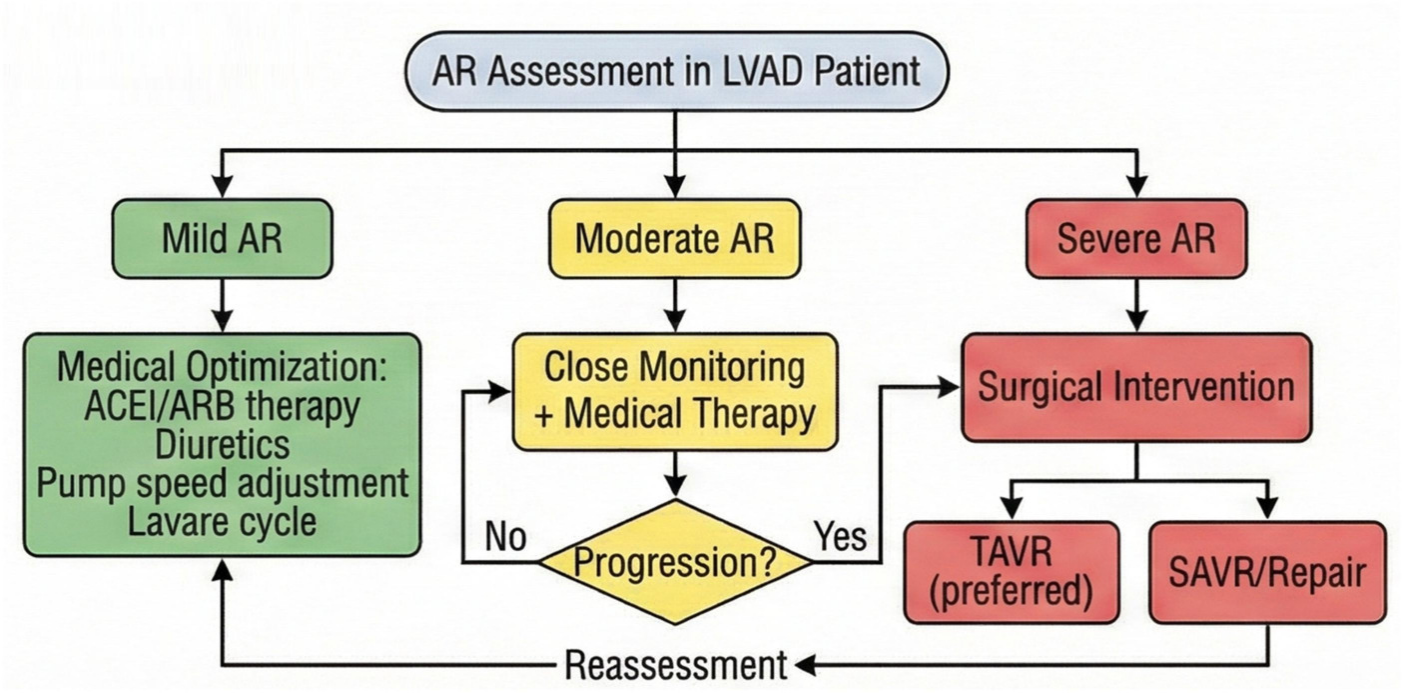
Proposed management algorithm for aortic regurgitation in LVAD patients, ranging from pharmacological optimization for mild AR to surgical intervention (TAVR/SAVR) for severe symptomatic AR.

The clinical management of AR after LVAD implantation still faces numerous challenges, and future efforts should focus on the following directions: developing LVADs with adaptive flow patterns to promote aortic valve opening through dynamic adjustment of pump speed and reduce valve load; exploring new technologies such as contrast echocardiography and myocardial strain imaging to improve the sensitivity of early AR diagnosis; developing specialized TAVR valves for LVAD patients and optimizing anchorage design; Establishing a long-term monitoring network encompassing AR progression, cardiac function evolution, and quality of life to provide evidence for optimizing treatment strategies. Innovations in pump design, including devices with physiological pulsatility algorithms that more closely mimic native cardiac function, hold promise for reducing the hemodynamic stress on the aortic valve and potentially decreasing AR incidence (85). Furthermore, the development of specialized TAVR valves designed specifically for non-calcified annuluses, incorporating novel anchoring mechanisms such as commissural alignment or atrial cuff designs, may expand treatment options for LVAD patients with significant AR who are not candidates for surgical intervention (86) (Figure 4).

## Conclusion

In summary, the management of AR following LVAD implantation requires comprehensive optimization across the entire chain of prediction, assessment, and intervention, achieved through multidisciplinary collaboration to enable personalized treatment. Preoperative risk stratification utilizing established predictors such as age, gender, body surface area, baseline valve status, and aortic root dimensions allows for targeted prophylactic interventions. Postoperative surveillance combining hemodynamic monitoring with multimodal imaging ensures timely detection of AR progression, while a stepwise treatment approach from medical optimization to transcatheter or surgical intervention provides a framework for individualized patient management. With ongoing technological innovation and accumulation of evidence, there is potential to significantly reduce the incidence of AR and improve long-term outcomes for patients with end-stage heart failure.

## References

[1] Savarese G Becher PM Lund LH Seferovic P Rosano GMC Coats AJS. Global burden of heart failure: a comprehensive and updated review of epidemiology. Cardiovasc Res. (2023) 118(17):3272–87. 10.1093/cvr/cvac013 35150240

[2] Yu Y Gupta A Wu C Masoudi FA Du X Zhang J Characteristics, management, and outcomes of patients hospitalized for heart failure in China: the China PEACE retrospective heart failure study. J Am Heart Assoc. (2019) 8(17):e012884. 10.1161/JAHA.119.012884 31431117 PMC6755852

[3] Varshney AS DeFilippis EM Cowger JA Netuka I Pinney SP Givertz MM. Trends and outcomes of left ventricular assist device therapy. JACC focus seminar. J Am Coll Cardiol. (2022) 79(11):1092–107. 10.1016/j.jacc.2022.01.017 35300822

[4] Truby LK Rogers JG. Advanced heart failure: epidemiology, diagnosis, and therapeutic approaches. JACC Heart Fail. (2020) 8(7):523–36. 10.1016/j.jchf.2020.01.014 32535126

[5] Yuzefpolskaya M Schroeder SE Houston BA Robinson MR Gosev I Reyentovich A The society of thoracic surgeons intermacs 2022 annual report: focus on the 2018 heart transplant allocation system. Ann Thorac Surg. (2023) 115(2):311–27. 10.1016/j.athoracsur.2022.11.023 36462544

[6] Dimarakis I Callan P Khorsandi M Pal JD Bravo CA Mahr C Pathophysiology and management of valvular disease in patients with destination left ventricular assist devices. Front Cardiovasc Med. (2022) 9:1029825. 10.3389/fcvm.2022.1029825 36407458 PMC9669306

[7] John R Mantz K Eckman P Rose A May-Newman K. Aortic valve pathophysiology during left ventricular assist device support. J Heart Lung Transplant. (2010) 29(12):1321–9. 10.1016/j.healun.2010.06.006 20674397

[8] Bouabdallaoui N El-Hamamsy I Pham M Giraldeau G Parent MC Carrier M Aortic regurgitation in patients with a left ventricular assist device: a contemporary review. J Heart Lung Transplant. (2018) 37(11):1289–97. 10.1016/j.healun.2018.07.002 30197211

[9] Gasparovic H Kopjar T Saeed D Cikes M Svetina L Petricevic M *De novo* aortic regurgitation after continuous-flow left ventricular assist device implantation. Ann Thorac Surg. (2017) 104(2):704–11. 10.1016/j.athoracsur.2017.01.114 28483150

[10] Tanaka A Kitahara H Onsager D Song T Raikhelkar J Kim G Impact of residual valve disease on survival after implantation of left ventricular assist devices. Ann Thorac Surg. (2018) 106(6):1789–96. 10.1016/j.athoracsur.2018.06.075 30148976

[11] Shad R Kaiser AD Kong S Fong R Quach N Bowles C Patient-specific computational fluid dynamics reveal localized flow patterns predictive of post-left ventricular assist device aortic incompetence. Circ Heart Fail. (2021) 14(7):e008034. 10.1161/CIRCHEARTFAILURE.120.008034 34139862 PMC8292193

[12] Hata H Fujita T Ishibashi-Ueda H Nakatani T Kobayashi J. Pathological analysis of the aortic valve after long-term left ventricular assist device support. Eur J Cardiothorac Surg. (2014) 46(2):193–7. 10.1093/ejcts/ezt559 24335262

[13] Truby LK Garan AR Givens RC Wayda B Takeda K Yuzefpolskaya M Aortic insufficiency during contemporary left ventricular assist device support: analysis of the INTERMACS registry. JACC Heart Fail. (2018) 6(11):951–60. 10.1016/j.jchf.2018.07.012 30384913 PMC6217859

[14] Singh R Strom JA Ondrovic L Joseph B VanAuker MD. Age-related changes in the aortic valve affect leaflet stress distributions: implications for aortic valve degeneration. J Heart Valve Dis. (2008) 17(3):290–8; discussion 299. 18592926

[15] Wingate S. Cardiovascular anatomy and physiology in the female. Crit Care Nurs Clin North Am. (1997) 9(4):447–52. 10.1016/S0899-5885(18)30237-5 9444167

[16] Cipolli JA Souza FA Ferreira-Sae MC Pio-Magalhães JA Figueiredo ES Vidotti VG Sex-specific hemodynamic and non-hemodynamic determinants of aortic root size in hypertensive subjects with left ventricular hypertrophy. Hypertens Res. (2009) 32(11):956–61. 10.1038/hr.2009.134 19713970

[17] Kagawa H Aranda-Michel E Kormos RL Keebler M Hickey G Wang Y Aortic insufficiency after left ventricular assist device implantation: predictors and outcomes. Ann Thorac Surg. (2020) 110(3):836–43. 10.1016/j.athoracsur.2019.12.030 31991135

[18] Nishida H Song T Onsager D Nguyen A Grinstein J Chung B Proximal ascending aorta size is associated with the incidence of *de novo* aortic insufficiency with left ventricular assist device. Heart Vessels. (2022) 37(4):647–53. 10.1007/s00380-021-01946-4 34585275

[19] Fine NM Park SJ Stulak JM Topilsky Y Daly RC Joyce LD Proximal thoracic aorta dimensions after continuous-flow left ventricular assist device implantation: longitudinal changes and relation to aortic valve insufficiency. J Heart Lung Transplant. (2016) 35(4):423–32. 10.1016/j.healun.2015.10.029 26632029

[20] Imamura T Kinugawa K. Preoperative prediction of aortic insufficiency during ventricular assist device treatment. Int Heart J. (2016) 57(1):3–10. 10.1536/ihj.15-250 26742702

[21] McKenzie NL Ward RP Nagele P Rubin DS. Preoperative β-blocker therapy and stroke or Major adverse cardiac events in major abdominal surgery: a retrospective cohort study. Anesthesiology. (2023) 138(1):42–54. 10.1097/ALN.0000000000004404 36227278 PMC9771981

[22] Imamura T Kinugawa K Nitta D Hatano M Ono M. Opening of aortic valve during exercise is key to preventing development of aortic insufficiency during ventricular assist device treatment. ASAIO J. (2015) 61(5):514–9. 10.1097/MAT.0000000000000247 25955152

[23] Cowger J Pagani FD Haft JW Romano MA Aaronson KD Kolias TJ. The development of aortic insufficiency in left ventricular assist device-supported patients. Circ Heart Fail. (2010) 3(6):668–74. 10.1161/CIRCHEARTFAILURE.109.917765 20739615 PMC3089421

[24] Uriel N Milano C Agarwal R Lee S Cleveland J Goldstein D Incidence and clinical correlates of de-novo aortic regurgitation with a fully magnetically levitated left ventricular assist device: a MOMENTUM 3 trial portfolio analysis. Eur J Heart Fail. (2023) 25(2):286–94. 10.1002/ejhf.2746 36404406

[25] Vidula H Takeda K Estep JD Silvestry SC Milano C Cleveland JC Jr Hospitalization patterns and impact of a magnetically-levitated left ventricular assist device in the MOMENTUM 3 trial. JACC Heart Fail. (2022) 10(7):470–81. 10.1016/j.jchf.2022.03.007 35772857

[26] Callington A Long Q Mohite P Simon A Mittal TK. Computational fluid dynamic study of hemodynamic effects on aortic root blood flow of systematically varied left ventricular assist device graft anastomosis design. J Thorac Cardiovasc Surg. (2015) 150(3):696–704. 10.1016/j.jtcvs.2015.05.034 26092505

[27] Kassi M Filippini S Avenatti E Xu S El-Tallawi KC Angulo CI Patient-specific, echocardiography compatible flow loop model of aortic valve regurgitation in the setting of a mechanical assist device. Front Cardiovasc Med. (2023) 10:994431. 10.3389/fcvm.2023.994431 36844719 PMC9945256

[28] Sayer G Sarswat N Kim GH Adatya S Medvedofsky D Rodgers D Kruse E The hemodynamic effects of aortic insufficiency in patients supported with continuous-flow left ventricular assist devices. J Card Fail. (2017) 23(7):545–51. 10.1016/j.cardfail.2017.04.012 28435003

[29] Mao JY Li DK Ding X Zhang HM Long Y Wang XT Fluctuations of driving pressure during mechanical ventilation indicates elevated central venous pressure and poor outcomes. Pulm Circ. (2020) 10(4):2045894020970363. 10.1177/2045894020970363 33282200 PMC7691920

[30] Raut MS Maheshwari A Desurkar V Bhavsar R. Rising central venous pressure: impending right-sided failure? Ann Card Anaesth. (2017) 20(4):440–1. 10.4103/aca.ACA_92_17 28994680 PMC5661314

[31] Nagy AI Venkateshvaran A Dash PK Barooah B Merkely B Winter R The pulmonary capillary wedge pressure accurately reflects both normal and elevated left atrial pressure. Am Heart J. (2014) 167(6):876–83. 10.1016/j.ahj.2014.01.012 24890538

[32] Kang G Ha R Banerjee D. Pulmonary artery pulsatility index predicts right ventricular failure after left ventricular assist device implantation. J Heart Lung Transplant. (2016) 35(1):67–73. 10.1016/j.healun.2015.06.009 26212656

[33] Haberka M Bałys M Gąsior Z Stasiów B. Aortic regurgitation and left ventricle remodeling on cardiac magnetic resonance and transthoracic echocardiography. Kardiol Pol. (2021) 79(9):965–71. 10.33963/KP.a2021.0047 34176113

[34] Otto CM Nishimura RA Bonow RO Carabello BA Erwin JP 3rd Gentile F 2020 ACC/AHA guideline for the management of patients with valvular heart disease: executive summary: a report of the American College of Cardiology/American Heart Association joint committee on clinical practice guidelines. Circulation. (2021) 143(5):e35–71. 10.1161/CIR.0000000000000932 33332149

[35] Sasai T Tokioka H Fukushima T Mikane T Oku S Iwasaki E Reliability of central venous pressure to assess left ventricular preload for fluid resuscitation in patients with septic shock. J Intensive Care. (2014) 2(1):58. 10.1186/s40560-014-0058-z 25705416 PMC4336121

[36] Karski JM. Transesophageal echocardiography in the intensive care unit. Semin Cardiothorac Vasc Anesth. (2006) 10(2):162–6. 10.1177/1089253206288991 16959743

[37] Flores AS Essandoh M Yerington GC Bhatt AM Iyer MH Perez W Echocardiographic assessment for ventricular assist device placement. J Thorac Dis. (2015) 7(12):2139–50. 10.3978/j.issn.2072-1439.2015.10.69 26793334 PMC4703697

[38] Voudris K Lesser J Sorajja P Hamid N. 3-dimensional multiplanar reconstruction with transesophageal echocardiography for alcohol septal ablation. JACC Case Rep. (2023) 24:102016. 10.1016/j.jaccas.2023.102016 37869218 PMC10589419

[39] Guglielmo M Rovera C Rabbat MG Pontone G. The role of cardiac magnetic resonance in aortic stenosis and regurgitation. J Cardiovasc Dev Dis. (2022) 9(4):108. 10.3390/jcdd9040108 35448084 PMC9030119

[40] Myerson SG d'Arcy J Mohiaddin R Greenwood JP Karamitsos TD Francis JM Aortic regurgitation quantification using cardiovascular magnetic resonance: association with clinical outcome. Circulation. (2012) 126(12):1452–60. 10.1161/CIRCULATIONAHA.111.083600 22879371

[41] Gulsin GS Singh A McCann GP. Cardiovascular magnetic resonance in the evaluation of heart valve disease. BMC Med Imaging. (2017) 17(1):67. 10.1186/s12880-017-0238-0 29284450 PMC5747097

[42] Zou H Jiang Y Huang H Elkoumy A Wang X Zhu J Automated and quantitative assessment of aortic root based on cardiac computed tomography angiography using a new deep-learning tool: a comparison study. Quant Imaging Med Surg. (2024) 14(12):8414–28. 10.21037/qims-24-650 39698729 PMC11651967

[43] Chourdakis E Koniari I Kounis NG Velissaris D Koutsogiannis N Tsigkas G The role of echocardiography and CT angiography in transcatheter aortic valve implantation patients. J Geriatr Cardiol. (2018) 15(1):86–94. 10.11909/j.issn.1671-5411.2018.01.006 29434630 PMC5803542

[44] Schechter MA Joseph JT Krishnamoorthy A Finet JE Ganapathi AM Lodge AJ Efficacy and durability of central oversewing for treatment of aortic insufficiency in patients with continuous-flow left ventricular assist devices. J Heart Lung Transplant. (2014) 33(9):937–42. 10.1016/j.healun.2014.04.017 24997496

[45] Letsou GV Musfee FI Lee AD Cheema F Delgado RM Frazier OH. Ten-Year survival with a continuous-flow left ventricular assist device and aortic valve closure. Tex Heart Inst J. (2020) 47(4):325–8. 10.14503/THIJ-19-7193 33472231 PMC7819440

[46] Ishigaki T Wakasa S. A simple closure method for a mechanical aortic valve in left ventricular assist device implantation. Gen Thorac Cardiovasc Surg. (2022) 70(7):677–9. 10.1007/s11748-022-01812-8 35391606

[47] Morgan JA Brewer RJ. Modified central closure technique for treatment of aortic insufficiency in patients on left ventricular assist device support. ASAIO J. (2012) 58(6):626–8. 10.1097/MAT.0b013e318271bc49 23103701

[48] Tulimat T Osman B Beresian J Sfeir P Borgi J. Management of a mechanical aortic valve during left ventricular assist device implantation in a previously replaced aortic root. Int J Artif Organs. (2022) 45(2):152–4. 10.1177/0391398821990667 33583241

[49] Robertson JO Naftel DC Myers SL Prasad S Mertz GD Itoh A Concomitant aortic valve procedures in patients undergoing implantation of continuous-flow left ventricular assist devices: an INTERMACS database analysis. J Heart Lung Transplant. (2015) 34(6):797–805. 10.1016/j.healun.2014.11.008 25511747 PMC4433438

[50] Feldman CM Silver MA Sobieski MA Slaughter MS. Management of aortic insufficiency with continuous flow left ventricular assist devices: bioprosthetic valve replacement. J Heart Lung Transplant. (2006) 25(12):1410–2. 10.1016/j.healun.2006.10.004 17178333

[51] Dranishnikov N Stepanenko A Potapov EV Dandel M Siniawski H Mladenow A Simultaneous aortic valve replacement in left ventricular assist device recipients: single-center experience. Int J Artif Organs. (2012) 35(7):489–94. 10.5301/ijao.5000102 22661109

[52] Hatano M Kinugawa K Shiga T Kato N Endo M Hisagi M Less frequent opening of the aortic valve and a continuous flow pump are risk factors for postoperative onset of aortic insufficiency in patients with a left ventricular assist device. Circ J. (2011) 75(5):1147–55. 10.1253/circj.CJ-10-1106 21378448

[53] Soria Jiménez CE Papolos AI Kenigsberg BB Ben-Dor I Satler LF Waksman R Management of mechanical prosthetic heart valve thrombosis: JACC review topic of the week. J Am Coll Cardiol. (2023) 81(21):2115–27. 10.1016/j.jacc.2023.03.412 37225366

[54] Kassaï B Gueyffier F Cucherat M Boissel JP. Comparison of bioprosthesis and mechanical valves, a meta-analysis of randomised clinical trials. Cardiovasc Surg. (2000) 8(6):477–83. 10.1177/096721090000800614 10996104

[55] Bidar E Folliguet T Kluin J Muneretto C Parolari A Barili F Postimplant biological aortic prosthesis degeneration: challenges in transcatheter valve implants. Eur J Cardiothorac Surg. (2019) 55(2):191–200. 10.1093/ejcts/ezy391 30541101

[56] Khan S Koerner MM Pae W Stephenson ER Weber H Brehm C Successful percutaneous transcatheter aortic valve replacement in multi-organ failure due to aortic bioprosthesis regurgitation in a patient with continuous-flow LVAD. J Heart Lung Transplant. (2013) 32(6):659–63. 10.1016/j.healun.2013.03.007 23701856

[57] Dhillon AS Jones BM Hodson RW Korngold EC. Transcatheter aortic valve replacement for severe aortic regurgitation in patients with a left ventricular assist device. J Invasive Cardiol. (2022) 34(5):E369–73. 10.25270/jic/21.00212 35343915

[58] Sassis L Kefala-Karli P Cucchi I Kouremenos I Demosthenous M Diplaris K. Valve repair in aortic insufficiency: a state-of-the-art review. Curr Cardiol Rev. (2023) 19(1):e270422204131. 10.2174/1573403X18666220427120235 35490315 PMC10201877

[59] Wong CHM Chan JSK Sanli D Rahimli R Harky A. Aortic valve repair or replacement in patients with aortic regurgitation: a systematic review and meta-analysis. J Card Surg. (2019) 34(6):377–84. 10.1111/jocs.14032 30953445

[60] Hynds MA Hayashi H Kurlansky P Zhao Y Vinogradsky AV Yuzefpolskaya M Medium-term outcomes of concomitant aortic valve repair in patients with continuous-flow left ventricular assist device. J Thorac Cardiovasc Surg. (2025) 169(6):1761–1769.e6. 10.1016/j.jtcvs.2024.05.016 38802043

[61] Mazzitelli D Stamm C Rankin JS Pfeiffer S Fischlein T Pirk J Leaflet reconstructive techniques for aortic valve repair. Ann Thorac Surg. (2014) 98(6):2053–60. 10.1016/j.athoracsur.2014.06.052 25468084

[62] Youssefi P El-Hamamsy I Lansac E. Rationale for aortic annuloplasty to standardise aortic valve repair. Ann Cardiothorac Surg. (2019) 8(3):322–30. 10.21037/acs.2019.05.13 31240176 PMC6562088

[63] Singhal AK Bang J Panos AL Feider A Hanada S Rankin JS. Concomitant aortic valve repair for aortic insufficiency and implantation of left ventricle mechanical support. J Card Surg. (2022) 37(7):2086–9. 10.1111/jocs.16547 35470913 PMC9320936

[64] Törngren C Jonsson K Hansson EC Taha A Jeppsson A Martinsson A. Medical therapy after surgical aortic valve replacement for aortic regurgitation. Eur J Cardiothorac Surg. (2023) 63(5):ezad042. 10.1093/ejcts/ezad042 36748999 PMC10196817

[65] Krishnarao K Krim SR. Management of hypertension in patients supported with continuous flow left ventricular assist devices. Curr Opin Cardiol. (2023) 38(4):281–6. 10.1097/HCO.0000000000001042 36927690

[66] Bhat V Kumar A Kalra A. Angiotensin-Converting enzyme inhibitors or angiotensin receptor blockers after transcatheter aortic valve replacement: a meta-analysis. JACC Adv. (2024) 3(5):100927. 10.1016/j.jacadv.2024.100927 38939627 PMC11198320

[67] Osmanska J Petrie MC Docherty KF Lee MMY McMurray JJV Campbell RT. Subcutaneous furosemide in heart failure: a systematic review. Eur Heart J Cardiovasc Pharmacother. (2025) 11(1):94–104. 10.1093/ehjcvp/pvae083 39520561 PMC11805693

[68] Beargie SM Tolbert L Tunney RK Cox ZL Gong W Zalawadiya S. Serial evaluation of loop diuretic efficiency following left ventricular assist device implantation. Int J Artif Organs. (2023) 46(10-11):555–61. 10.1177/03913988231193446 37646461

[69] Huang D Lacombe P Gulati G Couper GS Kawabori M Upshaw JN Association of diuretic requirement and right heart failure post-LVAD implantation. JHLT Open. (2024) 4:100082. 10.1016/j.jhlto.2024.100082 40144247 PMC11935456

[70] Imamura T Narang N. Implication of hemodynamic assessment during durable left ventricular assist device support. Medicina (Kaunas). (2020) 56(8):413. 10.3390/medicina56080413 32824131 PMC7466331

[71] Lai JV Muthiah K Robson D Prichard R Walker R Pin Lim C Impact of pump speed on hemodynamics with exercise in continuous flow ventricular assist device patients. ASAIO J. (2020) 66(2):132–8. 10.1097/MAT.0000000000000975 30913099

[72] Grinstein J Blanco PJ Bulant CA Torii R Bourantas CV Lemos PA A computational study of aortic insufficiency in patients supported with continuous flow left ventricular assist devices: is it time for a paradigm shift in management? Front Cardiovasc Med. (2022) 9:933321. 10.3389/fcvm.2022.933321 36337891 PMC9631475

[73] Uriel N Colombo PC Cleveland JC Long JW Salerno C Goldstein DJ Hemocompatibility-related outcomes in the MOMENTUM 3 trial at 6 months: a randomized controlled trial. Circulation. (2017) 135(21):2003–12. 10.1161/CIRCULATIONAHA.117.028303 28385948

[74] Mehra MR Uriel N Naka Y Cleveland JC Jr Yuzefpolskaya M Salerno CT A fully magnetically levitated left ventricular assist device. Final report. N Engl J Med. (2019) 380(17):1618–27. 10.1056/NEJMoa1900486 30883052

[75] Wever-Pinzon O Selzman CH Drakos SG Saidi A Stoddard GJ Gilbert EM Pulsatility and the risk of nonsurgical bleeding in patients supported with the continuous-flow left ventricular assist device HeartMate II. Circ Heart Fail. (2013) 6(3):517–26. 10.1161/CIRCHEARTFAILURE.112.000206 23479562

[76] Ando M Ono M. Concomitant or late aortic valve intervention and its efficacy for aortic insufficiency associated with continuous-flow left ventricular assist device implantation. Front Cardiovasc Med. (2022) 9:1029984. 10.3389/fcvm.2022.1029984 36457799 PMC9707693

[77] Noble S Mauler-Wittwer S. TAVR as an alternative to SAVR for pure native aortic regurgitation. Can J Cardiol. (2024) 40(2):316–25. 10.1016/j.cjca.2023.11.023 38016541

[78] Al Ahmad J Danson E. Transcatheter aortic valve implantation for severe chronic aortic regurgitation. J Clin Med. (2024) 13(10):2997. 10.3390/jcm13102997 38792538 PMC11122034

[79] Yoon SH Schmidt T Bleiziffer S Schofer N Fiorina C Munoz-Garcia AJ Transcatheter aortic valve replacement in pure native aortic valve regurgitation. J Am Coll Cardiol. (2017) 70(22):2752–63. 10.1016/j.jacc.2017.10.006 29191323

[80] Sawaya FJ Deutsch MA Seiffert M Yoon SH Codner P Wickramarachchi U Safety and efficacy of transcatheter aortic valve replacement in the treatment of pure aortic regurgitation in native valves and failing surgical bioprostheses: results from an international registry study. JACC Cardiovasc Interv. (2017) 10(10):1048–56. 10.1016/j.jcin.2017.03.004 28521923

[81] Fried JA Nazif TM Colombo PC. A new frontier for TAVR: aortic insufficiency in CF-LVAD patients. J Heart Lung Transplant. (2019) 38(9):927–9. 10.1016/j.healun.2019.06.024 31495409

[82] Dagher O Santaló-Corcoy M Perrin N Dorval JF Duggal N Modine T Transcatheter valvular therapies in patients with left ventricular assist devices. Front Cardiovasc Med. (2023) 10:1071805. 10.3389/fcvm.2023.1071805 36993995 PMC10040555

[83] Gondi KT Tam MC Chetcuti SJ Pagani FD Grossman PM Deeb GM Transcatheter aortic valve replacement for left ventricular assist device-related aortic regurgitation. The Michigan medicine experience. J Soc Cardiovasc Angiogr Interv. (2022) 2(1):100530. 10.1016/j.jscai.2022.100530 39132542 PMC11307432

[84] Carr MJ Smith SA Slaughter MS Pahwa S. Managing valvular pathology during LVAD implantation. Indian J Thorac Cardiovasc Surg. (2023) 39(Suppl 1):101–13. 10.1007/s12055-023-01567-8 37525709 PMC10387021

[85] Selzman CH Madden JL Healy AH McKellar SH Koliopoulou A Stehlik J Bridge to removal: a paradigm shift for left ventricular assist device therapy. Ann Thorac Surg. (2015) 99(1):360–7. 10.1016/j.athoracsur.2014.07.061 25442985 PMC4283551

[86] Thourani VH Kodali S Makkar RR Herrmann HC Williams M Babaliaros V Transcatheter aortic valve replacement versus surgical valve replacement in intermediate-risk patients: a propensity score analysis. Lancet. (2016) 387(10034):2218–25. 10.1016/S0140-6736(16)30073-3 27053442

